# 21083 Perceptions on the Role of Physical Therapy Providers for Falls Prevention: A Qualitative Investigation

**DOI:** 10.1017/cts.2021.723

**Published:** 2021-03-30

**Authors:** Jennifer L. Vincenzo, Susan Kane Patton, Leanne L. Lefler, Jason R. Falvey, Pearl A. McElfish, Geoffrey Curran, Jeanne Wei

**Affiliations:** 1 University of Arkansas for Medical Sciences; 2 University of Arkansas; 3 University of Maryland

## Abstract

ABSTRACT IMPACT: Being explicit about the prevention of falls throughout an older adults’ episode of care may further help reinforce the role of physical therapy providers in falls prevention and improve dissemination of this knowledge. OBJECTIVES/GOALS: The purpose of this study was to determine older adults’ awareness of and perspectives about the role of physical therapy providers for falls prevention and determine potential barriers and facilitators to utilization of preventive rehabilitation services METHODS/STUDY POPULATION: We used a qualitative descriptive phenomenological approach to emphasize participants’ perceptions and lived experiences. Four focus groups were conducted with 27 community-dwelling older adults (average age = 78 years). Focus groups were recorded, transcribed, condensed, and coded using thematic analysis. RESULTS/ANTICIPATED RESULTS: Surveys indicated 37% of participants experienced a fall in the last year and 26% reported suffering an injury. Four main themes and six subthemes surrounding older adults’ perceptions of physical therapy providers’ roles for falls prevention emerged: (1) Awareness of Falls Prevention (subthemes: I Don’t Think About It, I Am More Careful); (2) Being Able to Get Up from the Floor; (3) Limited Knowledge about the Role of Physical Therapy Providers in Falls Prevention (subtheme: Physical Therapy Services are for After a Fall, Surgery, or for a Specific Problem); and 4). Barriers to Participating in Preventive Physical Therapy Services (subthemes: Perceived Need and Costs, Access Requires a Doctor’s Prescription). DISCUSSION/SIGNIFICANCE OF FINDINGS: Older adults lack awareness about the role of physical therapy services in falls prevention, perceiving services are only to treat a specific problem or after a fall. Physical therapists should be explicit about the role of physical therapy in falls prevention for all older adults undergoing rehabilitation, regardless of the reason.

